# Significance of the Hepatic Falciform Artery

**DOI:** 10.7759/cureus.16440

**Published:** 2021-07-17

**Authors:** Sanjiv Gray, Latha Ganti

**Affiliations:** 1 Surgery, Lakeland Regional Medical Center, Lakeland, USA; 2 Emergency Medicine, Envision Physician Services, Plantation, USA; 3 Emergency Medicine, University of Central Florida College of Medicine, Orlando, USA; 4 Emergency Medicine, Ocala Regional Medical Center, Ocala, USA; 5 Emergency Medicine, HCA Healthcare Graduate Medical Education Consortium Emergency Medicine Residency Program of Greater Orlando, Orlando, USA

**Keywords:** hepatobiliary surgery, ct hepatic arteriography, hepatic artery chemoembolization, chronic hepatitis, hepatic falciform ligament artery

## Abstract

The authors present a case that highlights the anatomy of the hepatic falciform artery and describes its importance. The hepatic falciform artery is an anatomic variant that arises from the hepatic vasculature and provides arterial communication between the abdominal wall and the liver. It is essential to identify its presence, especially when surgery or embolization is planned for that area.

## Introduction

The hepatic falciform artery runs an extrahepatic course in the falciform ligament. It may arise from the middle or left hepatic artery and provide blood supply around the umbilicus and anastomoses with branches of the internal thoracic and superior epigastric artery [1,2]. Albrecht von Hailer first described the HFA in 1753. The venous collaterals between the anterior abdominal wall and the liver are well appreciated, but the arterial communications are less recognized. This may be due to the low incidence of identifying a distinct HFA on angiography versus the presence of multiple small vessels supplying the falciform ligament. The HFA may run as a single vessel or bifurcate early and run as two vessels in the falciform ligament [3].

An HFA has implications for surgeons and interventionalist during abdominal surgery, especially hepatobiliary surgery, where the proper identification of structures is crucial for safe surgery. The presence of aberrant or accessory vessels or ducts makes identification more difficult. A careful review of preoperative radiologic studies is critical for the anticipation of the anatomy. The HFA can serve as a collateral vessel in patients with severe mesenteric vascular occlusive disease by supplying the hepatic artery via retrograde filling and mesenteric circulation [4,5]. The HFA is the second most common non-hepatic artery arising from the hepatic vasculature. The most common is the right gastric artery. The authors present a case of such a hepatic falciform artery.

## Case presentation

A 34-year-old female with a history of hypertension presented to the emergency department with a complaint of abdominal pain for two days. The pain started in the epigastrium then radiated to the right upper abdomen and her back. The pain was described as being sharp and associated with chills, nausea and vomiting. She denied any chest pain, shortness of breath, diarrhoea, bloody vomit, bloody stools, or urinary problems. She had prior episodes of biliary colic but had not seen a surgeon previously. Her blood pressure was 115/75 mmHg, pulse 82 beats per minute, temperature 37.1^0^C, and respiration of 18 breaths per minute. Her physical examination showed no acute distress, normal mental status, and abdominal tenderness in the right upper quadrant.

Laboratory tests showed sodium 136mmol/L, potassium 3.6mmol/L, chloride 102mmol/L, carbon dioxide 28mmol/L, BUN 14mg/dl), creatinine 0.94mg/dl, glucose 82mg/dl, calcium 8.7mg/dl. Liver function tests and lipase were normal. Complete blood count revealed a white blood cell count (WBC) of 11.8K/mm3, haemoglobin of 14.7gm/dL, and platelets of 316K/mm^3^. Urinalysis revealed 2+ leukoesterase, WBC 11-20/high power field, and rare bacteria. 

**Figure 1:**
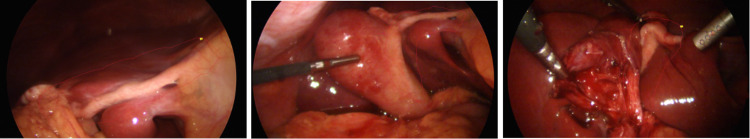
Intraoperative photos depicting the hepatic falciform artery (arrow)

Abdominal ultrasonography and computed tomography (CT) scan showed cholelithiasis and acute cholecystitis, and hepatic steatosis. The patient was admitted to the hospital and resuscitated with intravenous crystalloid solution and antibiotics. On hospital day one, the patient was taken for laparoscopic cholecystectomy. An acutely inflamed distended gallbladder with adherent omentum was noted (Figure 1a). There was an unusually large blood vessel and medial aspect of the gallbladder coursing toward the inferior liver edge and heading into the falciform ligament. The gallbladder was removed using caution to preserve the unusual blood vessel, and a critical view of safety was obtained. The aberrant blood vessel had several small branches going into the gallbladder (Figure 1b, 1c).

**Figure 2:**
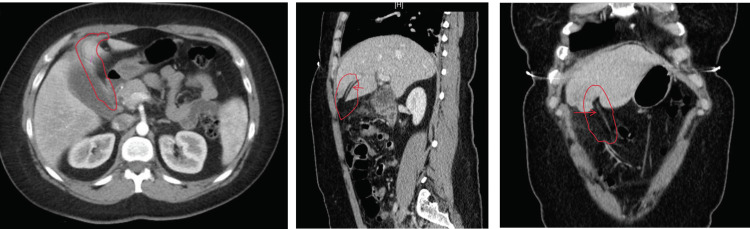
Computed tomography (CT) scans demonstrating the hepatic falciform artery (arrow)

The patient tolerated the procedure well, was extubated and transferred to the recovery room. On postoperative day one, the patient tolerated diet, the pain was controlled, and abdominal examination was unremarkable. The Jackson Pratt drain was removed, and the patient was discharged home. The patient followed up in the clinic after two weeks, at which time no immediate issues were identified. A review of the histopathology revealed moderate acute and chronic cholecystitis and cholelithiasis. On further review of her CT scan due to the intraoperative finding of the unusual blood vessel, the hepatic falciform artery could readily be appreciated (figure 2).

## Discussion

The HFA is seen infrequently during angiography, ranging from 2% to 25% of the population. The HFA is present in up to 68% of post mortem studies [1,5]. One study showed an incidence of 2% in a patient undergoing hepatic or celiac arteriography [6]. Another study compared the detection rate on computed tomography hepatic arteriography (CTHA) with that on angiography and dynamic CT and found that detection rates of FLA by angiography, dynamic CT, and CTHA were 37%, 10%, and 77%, respectively [1]. The calibres of FLA increased as the hepatic function deteriorated (p<0.0001). A 2017 study of 220 patients undergoing transarterial treatment for hepatic cellular carcinoma using C-arm CT hepatic arteriography (C-arm CTHA) showed a prevalence of 95% for the HFA. It documented the presence of Sappey’s superior artery in 22% [7].

The discrepancy between the incidence on angiography and postmortem studies is likely due to variation of techniques in regards to timing, a spatial resolution of angiography and the misidentification and overlapping of various structures in the hepatic hilum, vessel selection for angiographies such as celiac vs proper hepatic vs falciform artery and CT scan resolution, and vessel calibre. The presence of chronic hepatitis, cirrhosis, and mesenteric vascular occlusive disease is associated with the HFA. The detection rate may be higher in such patients [1,5].

Supraumbilical abdominal wall injury and rash has been described after transcatheter arterial chemoembolization (TACE) for hepatocellular carcinoma (HCC) and hepatic arterial infusion therapy through an indwelling catheter for metastatic liver tumours [1,3,5]. This complication is rare and likely due to hepatopetal flow from the epigastric arteries towards the liver. The identification and prophylactic embolization of the HFA are recommended before TACE, especially in patients with a prominent HFA [1,5,7]. Surgical ligation can be performed but is more invasive.

## Conclusions

Identification of the hepatic falciform artery, when present is important due to its communication with the anterior abdominal wall. Inadvertent chemoembolization of the vessel may lead to abdominal wall injury. Inadvertent injury to the vessel can occur intraoperatively when its presence is anticipated resulting in intraoperative bleeding. Thus, surgeons must be aware of the relevant anatomy and potential complications.
